# Health information management systems and practices in conflict-affected settings: the case of northwest Syria

**DOI:** 10.1186/s12992-024-01052-w

**Published:** 2024-06-06

**Authors:** Reem Ladadwa, Mahmoud Hariri, Muhammed Mansur Alatras, Yasir Elferruh, Abdulhakim Ramadan, Mahmoud Dowah, Yahya Mohammad Bawaneh, Wassel Aljerk, Preeti Patel, Abdulkarim Ekzayez, Nassim El Achi

**Affiliations:** 1 Research for Health Systems Strengthening in Syria (R4HSSS), Conflict and Health Research Centre CHRC, Department of War Studies, King’s College London, 11 Gainsford Street, London, SE1 2NE UK; 2 Research for Health Systems Strengthening in Syria (R4HSSS), Health Information System (HIS) Unit, Gaziantep, Turkey; 3 Research for Health Systems Strengthening in Syria (R4HSSS), Syrian Board of Medical Specialties (SBOMS), Gaziantep, Turkey; 4 Assistance Coordination Unit (ACU), Gaziantep, Turkey; 5 World Health Organization (WHO), Gaziantep, Turkey; 6 Syria Public Health Network, London, UK

**Keywords:** Health information systems, Conflict, Northwest Syria, Health data management

## Abstract

**Background:**

In conflict settings, as it is the case in Syria, it is crucial to enhance health information management to facilitate an effective and sustainable approach to strengthening health systems in such contexts. In this study, we aim to provide a baseline understanding of the present state of health information management in Northwest Syria (NWS) to better plan for strengthening the health information system of the area that is transitioning to an early-recovery stage.

**Methods:**

A combination of questionnaires and subsequent interviews was used for data collection. Purposive sampling was used to select twenty-one respondents directly involved in managing and directing different domains of health information in the NWS who worked with local NGOs, INGOs, UN-agencies, or part of the Health Working Group. A scoring system for each public health domain was constructed based on the number and quality of the available datasets for these domains, which were established by Checci and others.

**Results & conclusions:**

Reliable and aggregate health information in the NWS is limited, despite some improvements made over the past decade. The conflict restricted and challenged efforts to establish a concentrated and harmonized HIS in the NWS, which led to a lack of leadership, poor coordination, and duplication of key activities. Although the UN established the EWARN and HeRAMS as common data collection systems in the NWS, they are directed toward advocacy and managed by external experts with little participation or access from local stakeholders to these datasets.

**Recommendations:**

There is a need for participatory approaches and the empowerment of local actors and local NGOs, cooperation between local and international stakeholders to increase access to data, and a central domain for planning, organization, and harmonizing the process. To enhance the humanitarian health response in Syria and other crisis areas, it is imperative to invest in data collection and utilisation, mHealth and eHealth technologies, capacity building, and robust technical and autonomous leadership.

**Supplementary Information:**

The online version contains supplementary material available at 10.1186/s12992-024-01052-w.

## Introduction

Reliable health information is a critical requirement for the management of health systems [1–3]. Effective healthcare provision depends largely on timely and reliable health information, especially in the context of public health emergencies and early recovery settings [1–3]. Health information systems enable the collection, storage, analysis, and dissemination of health-related data to support decision-making and policy formulation. However, in conflict-affected settings and limited resource settings, accessing quality health information is a challenge and has received little attention amidst the often chaotic and ad-hoc provision of humanitarian healthcare. These issues are exacerbated during protracted conflict, which further deteriorates the provision and quality of healthcare and services.

With the changing nature and duration of conflict, efforts have increased in the last decade to increase the quality, accessibility, and availability of health information to inform and guide health interventions and service delivery in such contexts [2, 4]. mHealth and eHealth technologies offer digital solutions to major issues faced in the paper-based management of health information systems. For example, a mobile health system was developed in the Democratic Republic of Congo to improve healthcare provision in conflict-affected regions, where the system enables timely data collection and analysis, information sharing across health professionals, and improved health service delivery to meet identified needs or service gaps [5]. The system comprises a secure online platform that permits health workers to gather real-time data, exchange information, and analyse trends. Its main objective is to optimize efficiency by automating data collection, empowering field workers to obtain information promptly and precisely at the patient’s location [5]. Moreover, this approach enables health workers to retrieve patients’ medical records and other health-related data promptly, thus facilitating information-sharing and analysis. This, in turn, enables health workers to gain a better understanding of their patients’ health status, access pertinent information concerning treatment guidelines, and provide more effective and comprehensive care [5]. 

**Fig. 1:**
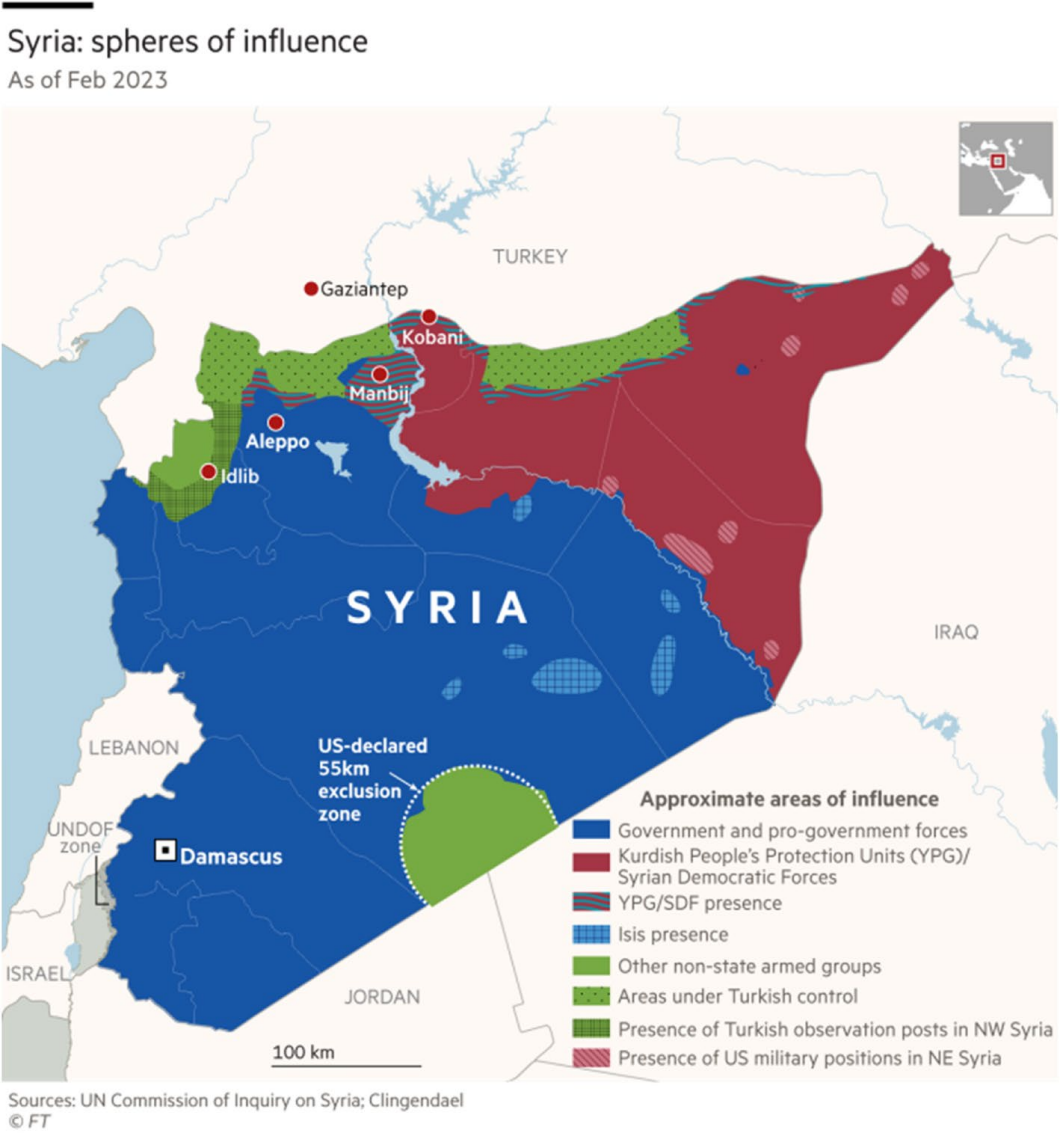
Forces and authorities of influence in the different regions of Syria. The top green areas are the focus of this study (Aleppo, Idlib and Hama). Source: This figure is copied from the Financial Times online article “Turkey and Syria face challenge to mend ties after years of ‘zero trust’” [8]

The Syrian conflict is a multisided conflict that involves various state and nonstate sponsored actors. It began in March 2011 as pro-democracy rallies and large-scale protests across Syria in line with the Arab Spring rallies in the Arab region. By 2012, multiple armed rebel groups had formed across the country, marking the beginning of the Syrian Civil War. Soon after, it developed into a proxy war, as foreign governments supported the different parties and groups at war inside Syria. The Syrian conflict is one of the worst humanitarian crises of the 21st century with the extent of reported civilian casualties in the last 10 years representing a staggering 1.5 per cent of the total population of the Syrian Arab Republic [6]. Moreover, out of the 22 million people who lived in Syria before 2011, more than 13 million had become refugees (5.5 million) or were internally displaced (6.8 million) [7]. The country is now divided into more than 8 areas that are under the influence of various state and nonstate and local and international groups [8]. Political, humanitarian, and economic conditions (95% of the population lives below the poverty line), continue to impact the daily lives of Syrians including their access to healthcare, as the country has multiple health systems and various healthcare providers each operating in silos, while still carrying the burden of the ongoing conflict (Fig. 1).

### Health information in Syria before and during the conflict

Pre conflict Syria had a relatively well-developed publicly funded health system, with a focus on secondary and specialised healthcare; however, health information management was weak and mainly primary [5–10]. The Ministry of Health (MoH) Information Department had a paper-based Health Management Information System (HMIS), designed to collect and manage health data from healthcare facilities across the country, including data on health service utilisation, health conditions, and service outcomes [5]. The MoH’s Information Department was responsible for analysing the data collected through HMIS and other sources, to support policy formulation [5]. Nevertheless, informants questioned both the availability and quality of these data, as well as its role in decision-making and policy formulation in the health system in Syria before the conflict. In the few years before the conflict, the MoH had introduced electronic-based HMIS to a very limited number of health facilities and disease-specific services [5, 10]. For example, the management of communicable diseases involved a combination of paper-based records and electronic systems. The MoH developed a reporting mechanism for communicable diseases, the Surveillance System for Communicable Diseases, which was utilised to track and respond to outbreaks of communicable diseases, and to provide information for public health planning and policy-making [10]. On the other hand, non-communicable diseases (NCDs) were typically managed in the public sector through paper-based records. Nevertheless, a considerable percentage of NCD patients sought medical care from private clinics, some of which employed electronic data systems to oversee NCD-related information [10]. 

Patient records in pre-conflict Syria were predominantly managed through paper-based records and stored in filing cabinets and paper folders, using arbitrary archiving methods inside healthcare facilities [11]. Healthcare providers managed these records without appropriate central management [11]. Additionally, communication and information sharing among healthcare providers were primarily performed through verbal communications and handwritten notes [11]. This constrained the ability to track patient outcomes and the quality of healthcare [5, 10, 12]. Despite the limited usage of electronic records in Syria, there was a gradual interest in applying such technologies in health information management [11, 13]. This has mainly emerged in private clinics and health facilities that developed their own electronic information systems to collect, manage, store, and retrieve records [11, 14]. However, the conflict hindered the implementation of such systems in the public health sector and severely limited the possibilities of mHealth and eHealth in health information management [5, 9–12, 14]. 

The Syrian conflict caused severe damage to health information management and limited the availability and quality of health-related data [5, 9–11, 14]. Many healthcare facilities have been destroyed or damaged during the conflict, leading to the loss of records, and the security situation in many parts of the country has also made it difficult to collect and report data, particularly in non-government-controlled areas [11, 12, 14]. In addition, health data in non-government-controlled areas is limited due to the collapse of central governmental services and the population movement and displacement [11, 12, 14]. Health professionals and institutions have encountered challenges in assessing population needs, the availability and quality of healthcare services, and the impact of the conflict on the health system or evaluating the effectiveness of public health responses [9, 11, 12, 14, 15]. 

To address the operational requirements for the humanitarian response, non-governmental organisations (NGOs) had to introduce new data collection systems that did not depend on pre-existing or pre-conflict records [5, 11, 14, 16]. For example, Doctors of the World (Médecins du Monde – MdM) implemented the District Health Information System − 2 (DHIS2) developed by the World Health Organization (WHO), to support the provision of primary health care and sexual and reproductive health services they provide through 14 fixed clinics and 2 mobile ones in Aleppo and Idlib in Non-Government Controlled Areas [17]. In addition, in 2012, the WHO established the Early Warning Alert and Response System (EWARS) to rapidly detect and respond to signals that might indicate outbreaks and clusters of epidemic-prone diseases [17]. The EWARS now covers 1,100 sites including the Syrian Ministry of Health and mainly targets government-controlled areas [17]. Similarly, the Early Warning Alert and Response Network (EWARN), a health information system for the surveillance and monitoring of epidemiological diseases, was established through the WHO, UNICEF and the Center for Disease Control in the USA in 2013. The EWARN has focused mainly on areas outside the Syrian government control (including Non- Government Controlled Areas) [17]. Despite the development of such systems for data collection, analysis, and management, the data was only utilised internally in the organisations and institutions that developed them [11]. Moreover, as these systems were designed to fulfil specific operational and reporting objectives, such data were of limited value in regard to health planning and priority setting [14]. 

In conflict settings, it is crucial to enhance health information management to facilitate an effective transition toward sustainable post-conflict health systems [1, 4]. Focusing here on the current gridlock of the conflict in northwest Syria (NWS) characterized by insecurity, heightened uncertainty, and instability, there is a need for a robust effort towards Health System Strengthening (HSS). NWS was selected as a case study because it is a region that lacks a defined and centralized form of government and the current provision and management of services, including health services, depends on the efforts of scattered local and international NGOs. The conflict in the NWS, as well as in Syria, is currently transitioning to a recovery post conflict stage in various regions in Syria and is expected to expand to the entire country in the near future, as efforts are becoming more effective at ending the conflict and reaching peaceful agreements. Given that the majority of HSS intervention frameworks place significant emphasis on managing health information, it is essential to have a comprehensive understanding of the present state of health information management prior to embarking on such investments. This study aims to develop a baseline understanding of the health information availability and practices in NWS and draws conclusions on ways forward in establishing and managing a sustainable short- and long-term health information system in NWS. It is important to note that findings of this study are not limited to conflict areas but extend to limited resource settings such as South Africa and Egypt. Hence, while the paper focuses extensively on conflict, the discussion and recommendations of the study are also applicable to and should be considered in limited resource settings.

### Health information system in low resource settings

HIS is an interrelated system offering easy access to patients’ medical records, reduced costs and time, and increased the production of evidence based healthcare interventions, research, and policy. In today’s digitalized world, HIS also offers comprehensive real time data for the design and development of quality healthcare applications, and allowed better accountability and healthcare government. A large portion of the literature we reviewed focused HIS for hospitals, specifically services related to surgery and pathology resulting in a gap in literature on HIS for less sophisticated healthcare facilities such as mobile clinics and primary healthcare centres that are mostly available in low resources settings [13]. There also seems to be a lack of literature on HIS from a delivery point of view. In some of the articles that focused on the delivery of HIS, website application were widely used which contradicts the increasingly available mobile based applications [13, 18–20]. Some articles argued that this contradiction is due to fear of data leakage and concerns over users’ data privacy [13, 18–20].

For papers that focused on the technicalities, designing user-friendly and secure HIS interfaces seemed to be a common challenge. In their paper, Zahabi et al. offer a set of standards to be followed for the development of user friendly and secure HIS platforms and interfaces [21]. Another common technical challenge in low resource settings was the frequent power and internet outage, which becomes specially an issue with the use of a computer, based or web based systems. Some articles propose relying on mobile applications for data entry and collection in such settings to overcome power and internet outages [22]. On challenges related to users, Younge et al. proposed a number of training methods that HIS end users (data entry officers and/or healthcare staff) could benefit from and suggests that multiple training approaches need to be used during the implementation of HIS applications and interfaces [23].

Lastly, the majority of reviewed literature reported challenges on the quality of HIS data that poses restrictions on data usage and techniques adoption such as adopting machine learning in healthcare predictions. Where the quality of entered data is problematic, following standardized codes such as ICD11 and ICPC in recording data would overcome the quality challenge [13, 24, 25]. When data is incorporated with other systems, following common information standards such as HL7 FHIR would be important [13, 26, 27]. Where certain data parameters are missing, automation and data driven machine-learning algorithms could be developed to use the available data to make predictions and useful healthcare outcomes. For example, Tutsoy and Tanrikulu designed a machine-learning algorithm to produce data driven prediction on future pandemics using COVID-19 limited health data. Similarly, Hong and Li used a similar approach to make time varying estimation of COVID-19 pandemic; Pinter et al. used hybrid machine learning approach to predict Hungary COVID-19 cases, and Tuli et al. used machine- learning and cloud computing to predict the growth and trends of the COVID-19 pandemic [28–30].

## Methodology

### Health information domains in conflict settings: a conceptual framework

**Fig. 2:**
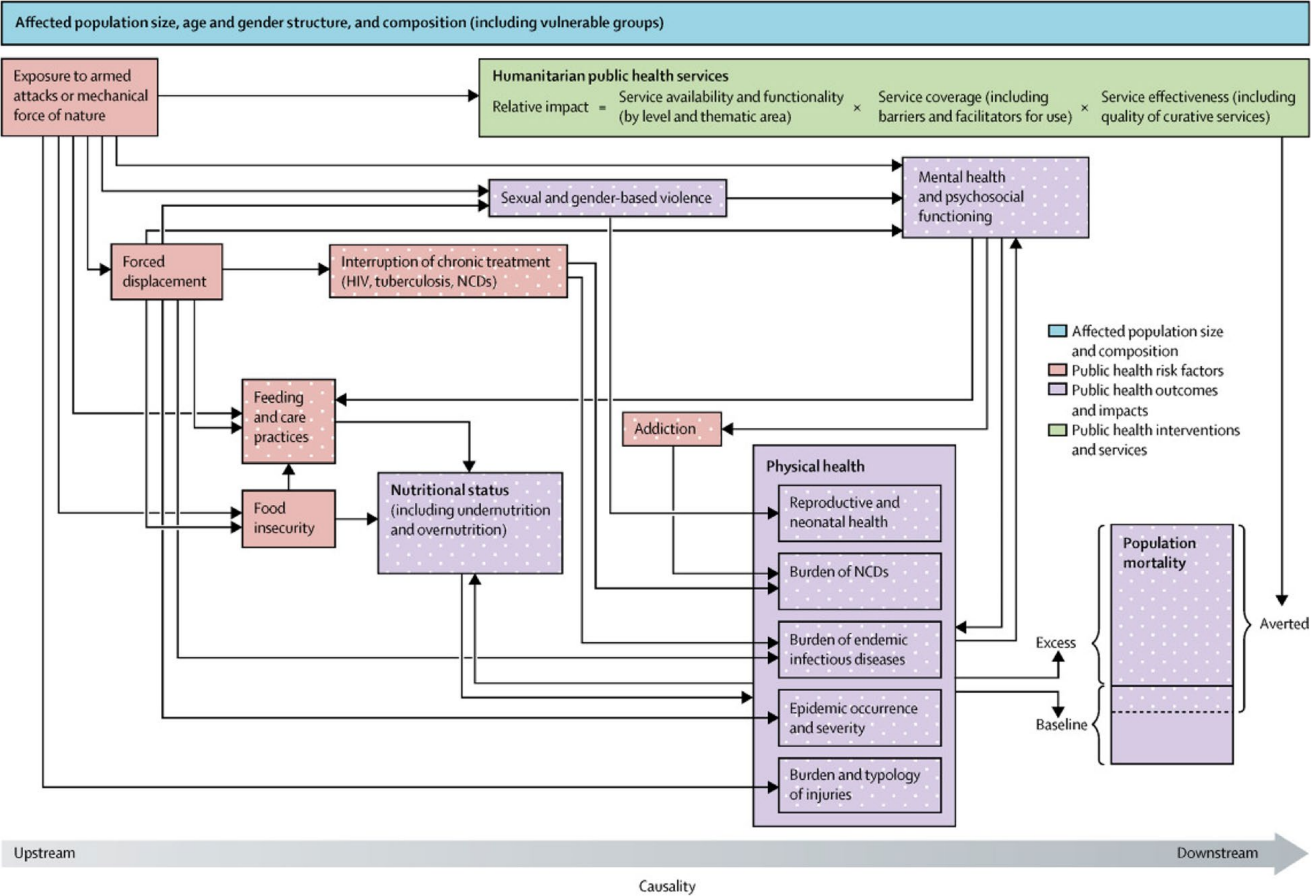
Domains of Public Health Information in Humanitarian Settings, Source: Checchi et al.: 2017 [31]

For this work, we used the conceptual framework for health information in humanitarian conflict settings that was published by Professor Francesco Checchi and colleagues in the Lancet in 2017. The group conducted a review of all available health information in conflicts taking place between 2010-2017 and found that data was only available in few contexts such as eastern Ukraine and Central African Republic [31]. Hence, they developed a baseline set of essential health information sets that should be available in humanitarian conflict settings. Authors categorized health information in conflict into various domains incorporating different aspects and usage of data. Domains include public health concerns during conflict and how they influence each other regarding key health outcomes (food security, nutritional status, morbidity…etc.) and effects on mental health, disability, and mortality. In addition, domains include humanitarian services such as Water, Sanitation, and Hygiene (WASH), nutrition, and healthcare, that seek to minimize the impact of conflict on the health of a conflict affected population. Figure 2 presents the domains for the minimal set of health information that needs to be available in conflict settings. For the purpose of this study, we relied on these domains to guide our research design and analysis.

### Data collection

We used a combination of questionnaires (Annex 1) and subsequent Key Informants Interviews (KIIs). The questionnaire, which was prepared on KOBO Toolbox, was built around the minimal set of health information in humanitarian settings [31]. It focused on the availability and the quality of data within the various domains of health information in the NWS context.

After filling the questionnaires in real-time using tablets having KOBO Toolbox software, the same respondent would then be interviewed in order to gain further insight into their areas of expertise, where the informants spoke about the strengths and gaps in the datasets they manage as well as the challenges they face in data collection and management.

Both the questionnaires and the KIIs were conducted whether face-to-face, or online, using Syrian Arabic between May 2022 and December 2022 and lasted for an average of 30 min. Purposive sampling was used to select 21 respondents involved in managing and directing different domains of health information in the region. Respondents were identified and selected through professional networks and featured key experts in their respective fields. They were carefully chosen to ensure that they had knowledge and experience related to the various domains of health information in NWS. They represented the following:

1. Local NGOs: Health information Unit [18], Syria Bright Future [19].
2. International NGOs: Relief International Humanitarian Needs Assessment Programme (RI- HNAP) [20], Union of Medical Care and Relief Organizations (UOSSM) [21], Physicians across Continents (PAC) [22], and Physicians for Human Rights (PHR) [23].
3. UN-based agencies and working groups: United Nations Population Fund (UNFPA), World Health Organization (WHO), the United Nations Office for the Coordination of Humanitarian Affairs (UN-OCHA), and the Health Working Group (HWG),

Once saturation as achieved, KIIs were then transcribed into Arabic then translated to English. All transcripts were anonymized using a unique identifier for each participant. We extracted the data generated from the questionnaires from KOBO Toolbox to Windows Excel for the analysis and NVivo 12 ® for data management and thematic analysis of that collected using the following KIIs.

### Thematic analysis for qualitative data

We adopted the 6-phase process described by Braun and Clarke [24]. First, two investigators (RL, MH) worked independently on coding transcripts line by line. Together, they discussed their coding approach to identify similarities and differences, thus avoiding bias, and created a thematic framework for data analysis. Second, the same investigators completed the open coding and started identifying emerging categories and themes. Members of the research team (AE, NE, RL and MH) met on several occasions to reflect on the findings. Finally, a complete narrative of the findings was generated and a validation meeting was conducted between research team and their advisors to present the findings and shape the recommendations and ensure the effective assessment of our findings. We then used the member checking approach to ensure confirmability and credibility. We used the consolidated criteria for reporting qualitative studies (COREQ): 32-item checklist for reporting the results, all components of the checklist were considered except the sharing of themes and transcribed data with participates as the time to complete the study was limited and did not allow further discussions with participants and respondents (Annex two).

### Increasing rigor for qualitative data

To further increase rigor, we focused on achieving both credibility and reflexivity. Regarding credibility, all discussions, were audio recorded, transcribed verbatim, accurately translated into English as necessary, and utilized as the main data repository. Once reaching data saturation, a decision was made by all team members to cease data collection. As for reflexivity, and to limit biases, all team members were involved in the analysis and interpretation of the results.

After analysing the collected data, we provided a scoring system (from 0 to 4) of the major public health information domains available in NWS. The scoring was based on the number and quality of available databases and sources for each domain category (Annex three).

### Quality of available data sets

The quality of datasets was determined based on the approach developed by Meredith Zozus and others to assess the data quality for healthcare systems [25]. Four dimensions of assessment are used: Data completeness, which assesses if the necessary data variables are present and sufficient data is available to calculate required outcome variables; data accuracy where the data is compared to other available data published by other institutions/organizations or compared to a confirmed range of expectations based on literature; data consistency across sites and facilities; and the frequency of updating the databases to reflect the reality on the ground.

## Results

### Overall characteristics

**Fig. 3:**
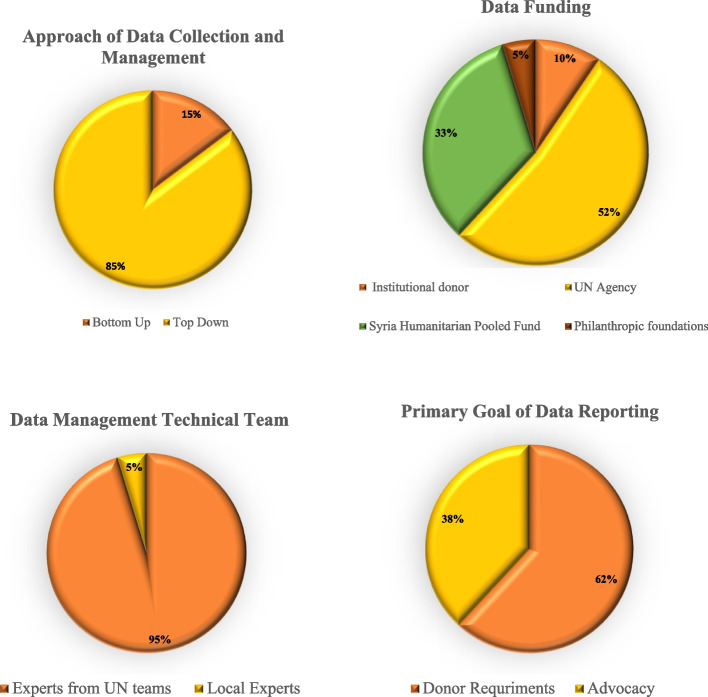
Characteristics of health information datasets included in this study. The top right figure refers to the methods and approach used by the organization in collecting and managing their health data. The top left figure is about the type of funding available for the organization to collect/manage health data. The bottom right figure refers to the data management team (if it is managed by an international team of experts of a local officer at the facility). The bottom left figure is about the facilities goal of collecting and managing health data

The study included health information and datasets from the health facilities and NGOs (local and international) that were accessible to the research team. As shown in Fig. 3, the majority of these datasets were designed and developed by international agencies, mainly UN ones such as WHO and IOM. The dominant approach to the design of data collection and management is top down where the dataset emerged from senior structures in an entity (such as UN agency or INGO) based on pre-allocated funding and data collection carried out by local officers. Similarly, all but one of the reviewed datasets were managed and directed by international expatriates with limited involvement of local staff. As such, more than half of the datasets and management systems were funded by UN agencies, mainly WHO and OCHA. In addition, about a third were funded through the Syrian Humanitarian Pooled fund (SHF), which is a country-based pooled fund led by the Humanitarian Coordinator for Syria and managed by the UN-OCHA. Since its inception in 2014, the SHF has been one of the primary sources of direct funding for Non-Governmental Organizations (NGOs) in Syria. For 2022 allocation, 76 per of funding is channelled to NGOs, of which 20 per cent only is allocated to national NGOs. Some data information systems were funded by Philanthropic foundations (e.g. Gates foundation Asfari Foundation, and Welcome trust) and Institutional donors (e.g. Foreign, Commonwealth & Development Office, European Civil Protection and Humanitarian Aid Operations, Office of U.S. Foreign Disaster Assistance…etc). In accordance with donor requirements and relations, the primary goal of data management was to satisfy the requirements of the funded projects and to evaluate their success and impact. Advocacy, especially towards an international audience for future funds, was also a major goal for collected data in NWS.

### Availability of the public health information domains in the NWS

#### Estimates of affected population size and composition

There is lack of available data on population size and composition in the NWS region, which poses a major gap in the health information system and impedes the ability of health actors to plan and evaluate effectively. The central civil registry in Damascus is the only known national source of population related information such as births, deaths, and age structure. The majority of health actors use estimations of UN led surveys for population size and health needs. This includes the Humanitarian Needs Overview (HNO), which is an annual exercise led by the UN-OCHA and is aimed at estimating population size and needs. The HNO relies mainly on self-reporting by humanitarian actors. The other source is the RI-HNAP, which is a joint UN assessment initiative that tracks population displacement and return movements. It conducts multi-sectoral assessments, and monitors humanitarian needs inside Syria. Local Syrian NGOs implement RI-HNAP with technical support from UN agencies. It employs community-based surveys as a methodology to estimate population size, population movements, and humanitarian needs. However, there are discrepancies and variations between these estimations and those provided by other local entities, such as local councils in each governorate.

Nevertheless, these are still some basic alternative sources of information that can be utilized to estimate population size and composition. These include data generated by the humanitarian heath response, such as vaccination data, Middle Upper Arm Circumference (MUAC) screening data and morbidity reports. For example, MUAC screening data is available in certain areas for all children aged five and under, due to mass screening initiatives conducted by some NGOs. This data can be used to estimate the number of children under five, which can then be extrapolated to generate estimations for the whole population using expected age structures. We found this to be one of the missed opportunities for health actors to validate the data provided by the surveys-based estimates.

### Information about public health risk factors

#### Exposure to armed attacks

Exposure to armed attacks was found to be among the most available data in the NWS. Health actors rely on various local and international databases and sources of information to be informed about recent safety and security related incidents that might affect their work. Some of the most cited databases and sources for exposure to armed attacks in the NWS are the International NGO Safety Organization (INSO), Physicians for Human Rights, and the Wartime and Post Conflict in Syria project of the Center for Operational Analysis and Research. In addition, NGOs rely on their internal safety and security analysis, with most of the NGOs informants reporting having a dedicated department for safety and security.

For attacks on healthcare facilities and professionals, international health actors and local ones were aware of the WHO led Surveillance System for Attacks (SSA). However, many NGOs reported limited trust and confidence in this system. Historically, the health cluster and its members developed an innovative mechanism for reporting called Monitoring Violence against Healthcare (MVH) [32]. The MVH was a customised reporting mechanism that used WhatsApp reporting with advanced data verification protocols and high engagement from local actors. However, WHO replaced the MVH in Syria by the SSA when it was launched globally in 2018 [33]. The SSA supports self-reporting by NGOs and health facilities for attacks and injuries through various forms of reporting including a user-friendly mobile application. Despite this, the mobile application is not in use in NWS and the system suffers from critical gaps that limit usability and trust among local health actors, as reported in Syria [33], and Ethiopia [16]. KIIs noted that data accuracy is an issue due to security restrictions, field visits are rarely carried out to confirm or validate violence allegations, which depend only on what is reported.

#### Sexual and gender-based violence (SGBV)

The United National Population Fund (UNFPA), which is the United Nations sexual and reproductive health agency, collected and managed a set of SGBV data in NWS. Additionally, the KIIs reported having systems in place for SGBV cases. The utilization of these systems, however, might be limited due to cultural barriers in some areas in NWS. Additionally, these internal reporting systems are not combined in a national level database and are not linked to local communication channels with local authorities or local communities. In addition, a major gap in these systems is that SGBV cases are reported only if they have been directed to a case management system. Furthermore, interviewees noted that the available SGBV database does not reflect the realities or extent of violence mainly due to under-reporting and lack of details in reported cases.

### Food security and nutritional status

Direct data on food security in the NWS was not available; however, some indicators were available through community-based surveys, feeding practice surveys, and internally displaced people’s health surveys and humanitarian action assessments. The assessments and surveys are usually conducted by NGOs that work in the sectors of food security and livelihood. Only some of these reports are accessible publicly. The frequency of these reports also seems to be fluctuating and follow only operational or donor reporting requirements. Local authorities do not seem to have accurate data on agriculture production of household livelihoods. These two areas could be filled by household surveys or community focus groups to make this information available.

The availability of data on nutritional status for internally displaced people seems to be more comprehensive and available. This is through an electronic monitoring tool developed by Rapid response team on KOBO, a software for remote data collection. This data was accurate for a large number of IDPs, however, follow up of cases was limited due to limited internet connectivity in the field and in office sites. Interviewees also noted that trained staff move between different NGOs and hence there is a need to train new recruits to sustain data collection, which is time-consuming and expensive.

### Information about public health services

#### Service availability and functionality

**Fig. 4:**
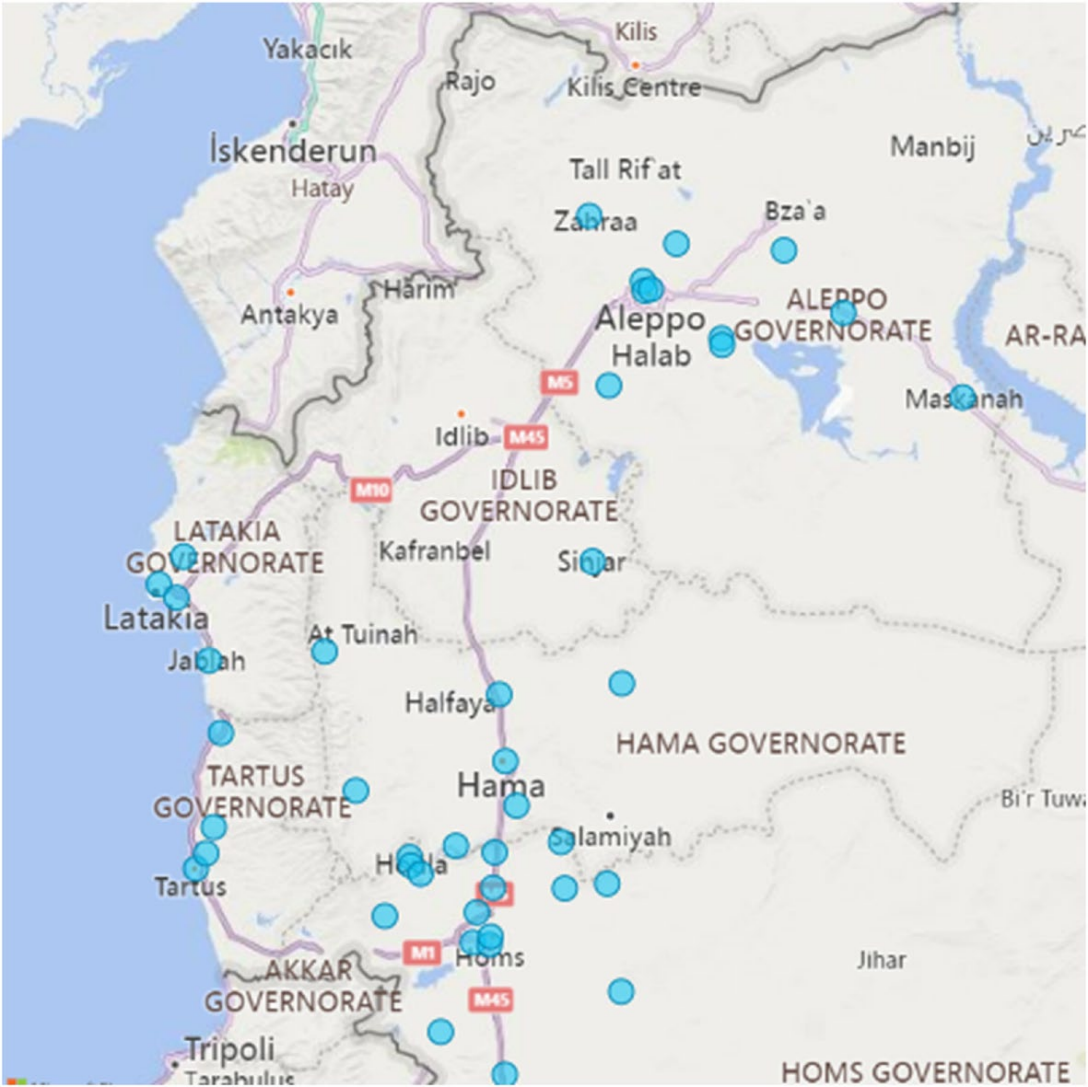
Distribution of health facilities in the NWS as per the 4WS reporting - source: HWG - NOV2022

Considering the absence of a national entity that manages health information in the NWS, the HWG plays a central role in health information management and dissemination, especially in relation to service availability, functionality and coverage. The HWG does this through three main data tools: the 4Ws (What, where, who, when) database, the mapping tool for health facilities, and the HWG monthly bulletin. However, all of these tools rely on self-reporting by NGOs, which raises questions on data completeness, accuracy, and reliability. On the positive side, the HWG uses indirect channels to communicate key findings of this data with local health authorities to inform their planning and implementation. Another example of good practice is where NGOs sign Memorandum of Understanding (MOU) with the local health committee where the health facility is based. Such practice was utilized by Relief International. As part of this MOU, RI shares monthly updates on service availability and functionality as well as some data on health outcomes with the local health committees. This is essential to involve local communities in health planning, implementation, and sustainability of services (Fig. 4).

As per the HWG’s mapping tool for health facilities in the NWS, there are a total of 63 health facilities in NWS as of November 2022. This number includes both international and local NGO run and facilities. The mapping tool also links the number of health facilities to the estimated number of populations in each sub-district in the NWS. This is especially useful in planning for resource allocations to reduce gaps in healthcare coverage.

Another tool is the Health Resources Availability Mapping System (HeRAMS), which is a routine exercise conducted on a quarterly basis in humanitarian crises led by the WHO and the health cluster. This exercise, however, is very limited in NWS where we found considerable difference between the reported numbers of health facilities in the recent HeRAMS report (HeRAMS – 2022) and the numbers from the HWG. These discrepancies can be attributed to the very limited presence of the WHO in the NWS as a leading agency for the HeRAMS exercise.

#### Service coverage

Data on coverage of health services is very limited in the NWS. While the 4Ws gives an estimation for the distribution of the locations of health services, there is no information on the actual coverage of these services. WHO established a program that identifies workload and gaps in service coverage in their partner primary health care centres in NWS, which provides some information on data coverage in the primary health care facility(s) they collaborate with. Vaccination registries on measles, polio, and COVID-19 provide some information about service coverage as well. However, this domain suffers from inaccurate population size which limits the measurement of service coverage and funds to establish and maintain such databases are limited and fluctuating. In addition, coverage of certain services, such as maternal health, family planning, and nutrition interventions is not fully known. This can be rectified through incorporating coverage outcomes within NGOs Routine Health Information Systems, and through coverage surveys for specific services (for example vaccination coverage surveys). This is crucial for the formulation of health policy and the provision of services to enable equitable access to health services on the one hand, and to identify key deficiencies in services on the other.

#### Service quality and effectiveness

Data on service quality and effectiveness was a key gap in this domain. We found no comprehensive datasets, or an agreed set of indicators related to health services quality on the NWS level. While some individual NGOs have various data systems and monitoring and evaluation mechanisms to monitor and report service quality, these systems are designed mostly for reporting purposes with limited clinical and health focus. The availability of such data depends on the reliability of the HIS used in each facility/NGO. Some systems do collect data on detailed health outcomes and allow for trend analysis of morbidities to identify gaps in quality. However, such systems are the exception in NWS, as most health actors use limited paper based HIS. Additionally, data on quality of services is only internal and is not shared externally. Limited analysis of dialysis, infection prevention and control, referral system, and health facilities datasets were also found. This provided information regarding quality of healthcare provision within these systems and their effectiveness in reaching their intended outcomes. However, lack of sustained funds and central governance of such data pose serious challenges. The HWG has recently developed a tool for Infection Protection and Control (IPC) assessment in health facilities. Such data tools that target specific elements of service quality can be expanded to cover some of the gaps in this type of data.

### Information about public health outcomes and impacts

#### Communicable diseases and public health emergencies

Data on communicable diseases, especially those that could cause outbreaks and pandemics, is relatively available in the NWS. There is currently one surveillance system for notifiable communicable diseases: EWARN. This surveillance is run by the Assistance Coordination Unit (ACU), which is an NGO based in Gaziantep/Turkey. The EWARN was established in June 2013 by the ACU. It is a health information system for surveillance and monitoring epidemiological diseases. The platform provides data on 14 different communicable, including Acute Bloody and Watery Diarrhoea, Acute Flaccid Paralysis, Acute Jaundice Syndrome, and Measles. EWARN uses the reporting forms, case definitions, and alert thresholds from the World Health Organization’s (WHO) field manual. Along with the weekly bulletins, EWARN issues a weekly surveillance of some notifiable diseases such as acute flaccid paralysis and water-borne diseases. Most of the EWARN reports are available publicly online via their website. The WHO is involved in providing technical support for this program, with EWARN being the main approved source of health data for northwest Syria by the WHO. The geographical coverage of the EWARN in the NWS is quite comprehensive. These systems managed to timely report and managed two polio outbreaks and have utilized social media to notify people of their test results and collect their registries. However, the low number of facilities and health staff per population as well as the continuous displacement and the precarious funding situation are challenges facing the management of data on communicable diseases in NWS.

In the wake of the Cholera outbreak in Kurdistan Iraq, the HWG recently developed a reporting tool for waterborne diseases. The tool was put into practice in August 2022 just before the Cholera outbreak hit the region in October 2022.

#### NCDs and mental health

The prevalence of Non-Communicable Diseases (NCDs) in the NWS is a critical area of knowledge gap. There is no information regarding the prevalence of key NCD morbidities such as diabetes and cardiovascular diseases. Lack of patients’ identification cards and lack of adherence to reporting mechanism pose a serious challenge to the health information system. NCD information was only available through WHO reports. Similarly, there is no standard reporting system in place for mental health morbidities with high impact on communities, such as suicidal thoughts, bipolar depression, and post-traumatic stress disorder despite reports by NGOs indicating high prevalence. Current challenges include lack of coordination among NGOs resulting in case duplication, stigma and taboo surrounding mental health resulting in under reporting (lack of accessibility of mental health services), lack of policy adherence to collected data and mental health indicators, and underfunding as funds are generally directed towards other priority areas such as communicable diseases.

#### Population mortality

The lack of mortality reporting in NWS poses a critical gap in health information. Neither facility-based mortality nor community-based deaths are adequately reported. No local authority or entity in the NWS has access to this data. KIIs with various NGOs revealed an absence of systems to identify the causes of death, except for medical notes issued by local doctors to be used by community members in registering deaths in the central registry. In addition, there is no common definition of reporting elements and procedure among the different health actors and lack of coordination and communication among the different health actors. The absence of identification cards is a major challenge resulting in case duplication and lack of data accuracy as entries are transferred from handwritten notes into computer-based registries in health facilities. Despite this, the case of COVID-19 mortality data collected in coordination with the EWARN team offers paths for future solutions on population mortality information systems.

## Discussion

Critical gaps in public health information in the NWS were found, especially in the areas of population size and composition, health risk factors (such as food security and livelihood), health service (such as coverage of services, and quality of services), and health outcomes (especially NCDs, mental health, and mortality). The following table summarizes the available information for the key health information domains in the NWS (Table 1):

**Table 1:** Available data on the main health information domains in the NWS

**Domain**	**Available information**	**Methods used**	**Agency**
**Population size and composition**	HNO	Self-reporting by humanitarian actors	UN-OCHA
	HNAP	Community based household surveys	Multiple Syrian NGOs and UN agencies
	Programmatic data	Nutrition screening, RMNCH services	MSF and other NGOs
**Exposure to armed attacks**	Security incidents data	Incident reporting, field focal points	INGO
	SSA database	Facility based surveillance	WHO
	Security analysis	Incidents reporting + security analysis	NGOs
**SGBV**	GBVIMS+	Analysis of program and HIS datasets.	UNFPA
**Food Security**	None	None	None
**Nutritional status**	MUAC Screening	Facility based and community based screening	PAC
**Service Availability**	HERMAS	Self reporting	WHO (Whole of Syria)
	4Ws	Self reporting	HWG
	Facility mapping	Self reporting	HWG
**Service Coverage**	EWARN and program reports	Prospective surveillance (EWARN.) + Population sample survey + Analysis of program data.	WHO
	Indicator reports (for funders)	Analysis of facility data	Multiple NGOs
**Service Quality**	M&E reports	Assessment based on Johns Hopkins dialysis task force	WHO
	WHO M&E reports on partner health facilities	Prospective surveillance (EWARN)	WHO
**CDs**	EWARN	Facility based surveillance + field focal points	ACU
	WHO CDs Outbreak reports	Population sample survey + Prospective surveillance	WHO
	Water born CDs dashboard	Self-reporting	HWG
	NCDs reports	District Health Information Software 2 (DHIS2) + Population sample survey + program data analysis	HWG
**Mental Health**	Suicide reports (advocacy reports)	Programming data analysis + population sample survey	Syria Bright Future
	Health Facility reports (Advocacy reports)	Programming data analysis	Union of Medical Care and Relief Organizations
**Mortality**	None	None	None

**Fig. 5:**
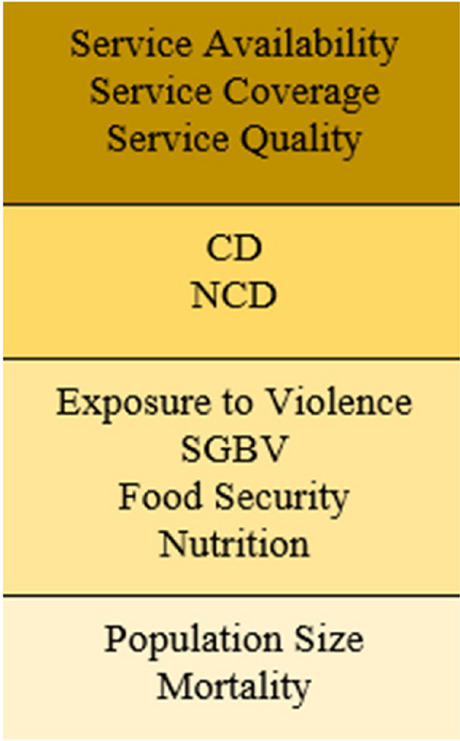
Availability and Quality of the Main Health Information Domains in the NWS according to our scoring system

Scores from 0 to 4 for each set of health information of these four domains were given based on the quality and number of available databases for each domain. The following diagram summarizes the scoring of the main public health information domains in the NWS. The colour scheme gets darker for domains with the most available information (Fig. 5).

In the absence of a national central body to manage health information in the NWS, international NGOs (mainly WHO and HWG) have become the central mechanism for data management and dissemination. To improve the availability and quality of health information, WHO and the HWG require increased technical support to strengthen the central mechanisms for data management, analysis, presentation, and dissemination and should work on building local capacities to sustain this function, specially that most of current systems are top down. Critical gaps in health data that humanitarian actors can help to fill include population size data, mental health, non-communicable diseases, and service quality. This can be done through:

1. Using programmatic data, from nutrition screening or HIS data form different facilities and NGOs, to estimate population size and composition.
2. Deploying a central reporting mechanism for major NCDs and mental health using the RHIS systems of NGOs to estimate prevalence and risk factors.
3. Developing a set of indicators for health service quality to standardize the level of services quality tracked by HWG and WHO.
4. Supporting health facilities to report and issue reports and forms on causes of death to better understand and estimate mortality data, especially on facility-based deaths.
5. Collaborating across sectors to complement health data on social determinants of health, food security, water, sanitation, and hygiene (WASH).

### Information sharing and a harmonized system

In general, NGOs provide information about services availability and coverage, with a number of them providing information on budgeting, resource allocation, procurement plans, and service beneficiaries [16]. INGOs and donor organizations provide public information through their websites on programs structures, available funds, decision making processes, and services distribution through their programs [16]. Information sharing and management is a real challenge in NWS and does not seem to be collected in an efficient way to support policy or intervention planning or implementation. NGOs collected data directly from the field or through other NGOs and local health facilities, while INGOs seemed to collect data through local projects they funded (project reports) and monitoring and evaluation reports.

In addition, in NWS, the local NGOs and INGOs do not have a harmonized system of health information collection, analysis, or sharing [9, 23–26, 34]. Data collection and processing is fragmented where each facility and institution has its own methodology of collecting and processing health data, with little information cross-sharing [9, 27, 30, 31, 35, 36]. Despite a poorly managed system of paper-based health information system in pre conflict Syria, there was available data on population denominators and concrete data that directed and guided policy planning and healthcare provision priorities and gaps [34, 37]. This was mainly attributed to the centralized role of the Syrian Ministry of Health that provided unified data collection forms across healthcare facilities (both public and private) and managed all of these data through a central team at the MoH [37]. However, in current NWS, data lives in silos and is mainly used in advocacy and funding efforts by both local and international NGOs. As such, there is little data sharing across different health actors in NWS.

At the initial stages of the Syrian conflict, reluctance in information sharing across institutions especially local and international was mainly due to security and authorization concerns. [26, 30, 31] Data on health facilities and programs was often used for targeting as one of the broad illustrated methods of healthcare weaponization in Syria, especially the NWS [26, 30]. As a result, strict information sharing protocols were implemented by INGOs which still pertain until today [26–30]. This lack of data sharing and data accessibility among the different healthcare actors in NWS is severely restricting coordination and strategic planning, which are essential especially in the recovery stage of healthcare system in NWS. Table 2 shows the available databases of health information on multiple domains at the ReliefWeb Response website, which is a specialized digital service of OCHA. There is a demonstrated difference between the amount of data ReliefWeb, HWG, and HIS have access to compared to accessible data of this study, which is a reflection on the overall landscape of health information sharing systems in NWS. There are a number of mutual databases that are shared between RW website and this paper findings, and a number of more concrete databases available for RW especially in relation to mortality and healthcare coverage. In a region that suffered over a decade of conflict, data on morbidity, mortality, healthcare provisions, and healthcare facilities are essential to support appropriate public health actions, identify the population healthcare needs, identify humanitarian interventions, and documenting conflict impacts on health and on civilians. [26–30, 38, 39] It is also essential for effective advocacy campaigns and funding campaigns to mitigate some of the conflict impacts on the health of civilians [25, 26, 40]. Most of the current data on war affected Syrians is on refugee populations in neighbouring countries in Jordan, Lebanon, and Turkey [9–14, 16–33, 36, 41, 42]. Substantives public health analysis for Syrian population in the NWS is lacking [9, 26, 28–31]. Health data on the conflict trapped population in NWS is essential and requires a collective effort and collaboration among the various local and international actors in NWS who have valuable data on population and health conditions of the NWS population.

**Table 2:** ReliefWeb health information data in NWS (including Whole of Syria – WoS)

**Category**	**Name**	**Responsible**
**4Ws**	4Ws HRP WHO Syria snapshot	HIS
	4Ws HRP health sector interactive dashboard	HIS
	WoS health cluster 4 W HRP	HIS and WoS
**COVID-19**	COVID-19 interactive dashboard (English version)	HIS
	COVID-19 interactive dashboard (Arabic version)	HIS and MoH
	WoS COVID-19 Update	HIS
	COVID-19 WHO and health sector response monitoring	HIS
	COVID-19 WHO Syria response monitoring infographic	HIS
	COVID-19 EPI Bulletin	HIS and surveillance and Immunization
**HeRAMS**	HeRAMS PHC	HIS
	HeRAMS PHC interactive dashboard	HIS
	HeRAMS Public Hospitals	HIS
	HeRAMS Public Hospitals interactive dashboard	HIS
	HeRAMS PHC report	HIS
	HeRAMS Public Hospitals report	HIS
	HeRAMS SARC report	HIS
	HeRAMS UNRWA report	HIS
**HNO & HRP**	HRP	WHO
	PMR (Periodic Monitoring Report), HRP	WHO
	HNO	WHO
**EWARS**	EWARS Syria Bulletin interactive dashboard	HIS
	WoS EWARS(N) Bulletin	HIS
	Al Hol mortality report	HIS
	EWARS Syria Bulletin	HIS
**Nutrition**	Neonatal Resuscitation	HIS and Nutrition
	Nutrition surveillance in Syria | Children under 5 years	HIS and Nutrition
	Integrated Management of Childhood Illness (IMCI)	HIS and Nutrition
	New Borne care at home	HIS and Nutrition
**Health sector**	Overview of capacity building events supported by sector	HIS
	Overview of rehabilitation activities supported by sector	HIS
	Health sector bulletin	HIS
	Health sector field directory	HIS
	Cluster coordination performance management (CCPM)	HIS
	Health sector assessment registry	HIS
	Health sector contact list	HIS
	Inventory of health sector projects	HIS
	Operational coverage by health sector	HIS
**Attacks**	Flash updates on attacks on health care	HIS
	Humanitarian Response	WHO

### E-Health and m-Health opportunities

The fragmented nature of the Syrian conflict poses an opportunity to develop and implement eHealth tools to overcome barriers of data collection and management [26, 30]. Investments in eHealth to facilitate automated transmission and exchange of health data has the potential to overcome HIS conflict barriers such as security and safety concerns and remote management [27, 30]. In Lebanon, for example, the Global Health Institute of the American University of Beirut, developed and implemented an eHealth project (*E-Sahha*) which delivers healthcare to underserved populations such as Palestinian and Syrian refugees in Lebanon [43]. The health tool and content were designed and developed in collaboration with experts at the Ministry of Public Health, as well as health workers and users of primary care centres [43–47]. Based on this collaboration, the tool targets health patients for information about their healthcare, health workers and providers for care follow-up, and the Ministry of Public Health for documentation and data management. The digital platform was launched in 2014, and to date it generated data on prevalence of non-communicable diseases and depression and forms the central house of refugee health and population data in Lebanon allowing for informed health policies and interventions [43–47]. 

**Fig. 6:**
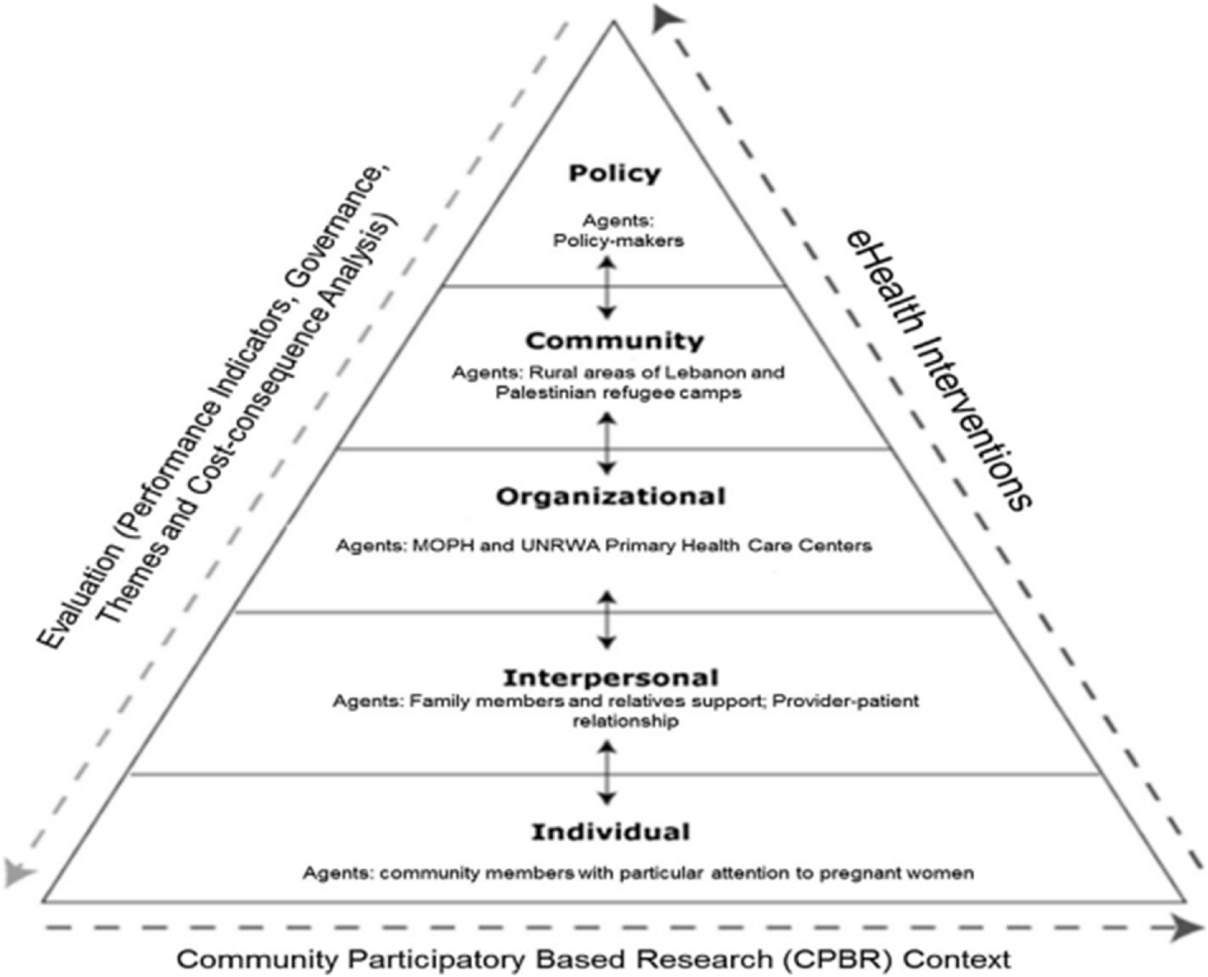
Conceptual framework of community based approach to mHealth design and development. Adapted from: Balca´zar et al. (2012)

The *E-Sahha* platform was developed through a community participatory approach, which was key for its success despite the complex environment it operates in (refuges in crisis affected Lebanon) [43, 46, 47]. The participatory approach is necessary for the success of any health information system tools and platforms in the Syrian case, to assure overcoming local barriers and to empower local actors who are key users of such platform [48]. The participatory approach is an ecological one and includes three key pillars: intervention, evaluation, and community participation as shown in Fig. 6 [48].

Such an approach could also be applied to data management platforms such as DHIS-2, which is currently used by local and international NGOs in NWS. DHIS-2 is an open-source information system with minimal hardware requirements. It aggregately stores routinely collected data across health facilities of a given country to facilitate analysis of health services and project health needs at a national level [49, 50]. DHIS-2 is strong in relation to data visualization and provides the option of carrying out certain operations offline, although Wi-Fi is needed for launching and for data transferring [49–51]. In addition, as it is an open source, it offers flexibility for users to modify the platform to meet their needs [49–52]. However, the use of DHIS-2 is challenging in the Syrian context due to concerns around data security, availability and quality of data, and the integrity of the health system [49–51]. As the DHIS-2 was developed on the premise of open data with granted access to all involved stakeholders which poses risks related to data security especially in the context of healthcare weaponization in the Syrian context [53, 54]. Furthermore, for sufficient reporting outcomes, data entered DHIS-2 needs to be in specific format and with a number of variables to comply with DHIS-2 modules [49–52]. Given the current fragmented and non-harmonized data collection in NWS, sufficient usage of DHIS-2 is challenging. The health information system in NWS developed in a fragmented manner during the conflict, resulting in lack of data sharing, data duplications, improper use of data and disruption in healthcare provision, which pose challenges to data entry and analysis in DHIS-2 [49–52]. Another key challenge is that launching and implementing DHIS-2 in most countries requires external consultants (from the University of Oslo or WHO) which is restricted especially in NWS where the political environment is instable and insecure [49–52]. Modifying the system to allow for independent launching and implementation is important in conflict settings, especially that in NWS. If such a modification is not possible, then allowing communication pathways between local actors and external consultants is essential.

However, it is important to consider the limitations of mHealth and eHealth applications as they rely on functional internet connection and infrastructure as well as available electricity and communication infrastructure, which are not always present in conflict and limited resource settings. While this study points out these limitations, it also recognizes some solutions such as satellite internet and solar powers, which could overcome significant infrastructure and connection challenges if applied and implemented in regions of interest.

### Empowering local agency

In NWS, local NGOs and health facilities are mainly responsible for managing health services for the population including internally displaced ones and needed to adapt to complex and new organizational mechanisms including new bilateral and multilateral actors such as international and humanitarian NGOs and UN and donor agencies with varying missions, mandates and agendas. [9, 11, 14, 16, 28, 29] Such a landscape resulted in a poorly coordinated and fragmented healthcare response in NWS and undermined local strategic plans and exiting local structures, which also reflected in HIS as explained earlier. [14, 28, 29] In addition, this landscape resulted in a general confusion over roles, responsibilities, and accountability, especially that multiple systems of hierarchy are in place in NWS from local to international organizations and institutions [26, 27, 35]. Therefore, empowerment and capacity building of local NGOs and actors working in the field is important to overcome these challenges. Almost all of the health datasets explored in this study were developed in a top-down manner, with minimal power of local actors beyond data collection. Most of the health information regarding Syrians focuses on Syrian refugee populations, with a limited information on the paralyzed health sector and the remaining resident population including internal displaced persons in NWS [26–29]. There is a need to include, empower, and build capacity and engagement of local actors in the process of data collection, management, analysis, and publication. Collection and management of data systems in humanitarian response in conflict settings and post conflict is proven to be more effective when local NGOs and health actors are involved with a large level of authority and leadership [16, 31, 34, 39, 41, 55]. Examples from Lebanon (*E-Sahha* platform), Palestine (*DHIS-2* platform), Afghanistan (*Aga-Khan*, and *IPath*) proves the efficacy of adopting an ecological local community approach towards HIS systems development and management. [16, 30, 34, 38, 39, 41, 49] A participatory approach to health information collection and management also aligns with the recent global momentum to move away from short hierarchal health projects in conflict towards long-term, development-oriented, and locally engaged health systems with coordination with external actors [33, 38]. 

### Limitations

One of the main limitations is perhaps that the findings may not be generalizable outside the NWS context, although a follow-up comparative study focusing on other areas in Syria would enable broader interpretations towards harmonisation of health information system in all of Syria [56]. Another limitation is that the study took place before the February 2023 earthquake that has had drastic consequences on the health system in NWS with all development activities being halted in favour of humanitarian support. However, this does not jeopardise the key results and findings of the study but rather highlight the need for more attention towards robust and sustainable solutions for improved health information systems in conflict and fragile settings. [12, 15, 36, 40, 43–48, 50, 51, 53–55, 57, 58].

## Conclusions and recommendations

Reliable and aggregate health information to support evidence-based policy and research to the health crisis in NWS is limited, despite some improvements made over the past decade. The conflict restricted and challenged efforts to establish a concentrated and harmonized HIS in NWS due to displacement, security concerns, fragmentation of leadership and responsible authorities, high levels of uncertainty, and political and physical barriers to aid delivery. The UN attempted to overcome some of these challenges and established systems such as EWARN and HeRAMS to act as a common data collection system in NWS. However, most of these systems are directed towards advocacy and funding efforts and are managed by external experts with little participation of local NGOs or Syrian experts at the leadership or development levels. This paper revealed several data collection and management challenges in the NWS context including duplication of cases and key activities, lack of leadership, poor coordination, limited information and data sharing, restricted and limited NGOs capacity, and continuous displacement in NWS. Findings of this paper raises key recommendations for HIS in NWS: *The need for participatory approach and empowerment of local actors and local NGOs; Corporation between local and international and increase accessibility; and a central domain for planning and organization and harmonization of the process.*

### Supplementary Information

Supplementary Material 1.

## Data Availability

The datasets generated and analysed during the current study are not publicly available due to them containing information that could compromise research participant privacy/consent but are available from the corresponding author on reasonable request.

## Abbreviations

NWS: North west Syria
NGO: Non governmental organization
INGO: International non governmental organization
UN: United nations
HIS: Health infromation system
EWARN: Early warning alert and response network
HeRAMS: The health resources and services availability monitoring system
MoH: The ministry of health
HMIS: Health management information system
NCDs: Non-communicable diseases
CDs: Communicable diseases
MdM: Doctors of the world (Médecins du Monde)
DHIS2: The district health information system -2
WHO: World health organization
EWARS: The early warning alert and response system
UNICEF: The united nations children's fund
HSS: Health system strengthening
WASH: Water, sanitation, and hygiene
RI- HNAP: Relief international humanitarian needs assessment programme
UOSSM: Union of medical care and relief organizations
PAC: Physicians across continents
PHR: Physicians for human rights
UNFPA: United nations population fund
UN-OCHA: United nations office for the coordination of humanitarian affairs
HWG: The health working group
SHF: The syrian humanitarian pooled fund
MUAC: Middle upper arm circumference
INSO: The international ngo safety organization
SSA: The who surveillance system for attacks
MVH: Monitoring violence against healthcare
SGBV: Sexual and gender-based violence
KIIs: Key informants interviews
IDPs: Internally displaced persons
MOU: Memorandum of understanding
RI: Relief international
IPC: Infection protection and control
ACU: Assistance coordination unit
MSF: Doctors without borders (Médecins Sans Frontières)
4Ws: Who, Where, When, doing What?
M&E: Monitoring and evaluation
GBVIMS: Gender-based violence information management system
WoS: Whole of syria
HNO: Humanitarian needs overview
HRP: Health response plan
SARC: The syrian arab red crescent
UNRWA: The united nations relief and works agency
PMR: Periodic monitoring report
IMCI: Integrated management of childhood illness
CCPM: Cluster coordination performance management
