# Supplementary material for: Health information management systems and practices in conflict-affected settings: the case of northwest Syria
Source: Global Health. 2024 Jun 6;20:45. doi: 10.1186/s12992-024-01052-w (PMC11155176; doi:10.1186/s12992-024-01052-w)
Supplement: Supplementary file 1 — Supplementary Material 1. [file 12992_2024_1052_MOESM1_ESM.docx]

**Annex 1: Questionnaire**

**What is your affiliation *(dropdown list)***

| WHO |
| --- |
| HNAP |
| Ocha |
| UNFPA |
| WFP |
| SIG |
| UNHCER |
| MPHSS TWG |
| NCD TWG |
| EWARN |
| PAC |
| UOSSM |
| PHR |
| SAMS |
| HIS Unit |
| Other (specify) |

**Common Questions for all interviewees:**

1. When was the dataset established?

*Please insert the year of establishment*

1. What is the type of funding the dataset receives? ***(one or more options)***
2. Syria Humanitarian Pooled Fund
3. Syria Recovery Trust Fund
4. UN agencies
5. Institutional donor (e.g. FCDO, ECHO, OFDA…etc)
6. Unrestricted funding from NGOs
7. Other ***(specify)***
8. What was the approach for establishing the dataset at the first place?
9. Bottom-up: i.e., the dataset emerged from field networks
10. Top-down: i.e., the dataset emerged from senior structures in an entity (such as UN agency or NGO) based on pre-allocated funding.
11. A collation of datasets: i.e., the dataset emerged from merging several datasets together.
12. Other ***(specify)***
13. What is the technical support the dataset receives? ***(one or more options)***
14. Experts from UN agencies
15. Experts from philanthropic foundations (e.g. Gates foundation)
16. Experts from International NGOs
17. Local experts.
18. No available technical support.
19. Other ***(specify)***
20. Methods used for data collection: ***(one or more options)***
21. Prospective surveillance
22. Population sample survey
23. Analysis of program data
24. Others. ***(specify)***
25. The use of technology in data collection: ***(one or more options)***
26. Kobo toolbox
27. DHIS2
28. Locally developed software (Please provide more details)
29. The use of social media (e.g. WhatsApp) (Give more details)
30. Other: ***(specify)***
31. How often are reports generated? ***(one or more options)***
32. Weekly
33. Monthly
34. Quarterly
35. Semi-annually
36. Annually, and
37. On time
38. Other ***(specify)***
39. To whom reports are disseminated: ***(one or more options)***
40. Internal reports
41. Doners
42. UN agency
43. National entities (Directorates)
44. Central HIS Unit
45. Other ***(specify)***
46. How do you utilize your reports? ***(one or more options)***
47. Fulfilling the donor requirements
48. Advocacy
49. Program design
50. Urgent decisions
51. Coordination purposes
52. Other: ***(specify)***
53. Please describe challenges at the different aspect of data management. ***(Minimum 3 challenges)***
54. Please tell stories of success.

**Questions specific to each domain:**

The questions here will investigate the methodologies available for each domain. We will assign 1 value for the used method or 0 value when it is not available. Also, if other methods not mentioned in the checklist are used, they will be written in a textbox.

|  |  |  | Comment |
| --- | --- | --- | --- |
| 1 | **Affected population**  **size and composition** | Community-based demographic surveillance |  |
|  |  | Residential structure tally and structure occupancy estimation |  |
|  |  | Vaccination or nutritional screening data combined with expected age structure |  |
|  |  | Other: ***(write in the column to the right)*** |  |
| 2 | **Exposure to armed attacks or mechanical force of nature** | Facility-based surveillance of injuries and attacks against health care |  |
|  |  | Retrospective survey of individual exposure to injury |  |
|  |  | Conflict analysis (tracking of media and other informant reports) |  |
|  |  | Other: ***(write in the column to the right)*** |  |
| 3 | **Sexual and gender-based violence** | Facility-based surveillance of sexual and gender-based violence cases |  |
|  |  | Retrospective survey of individual exposure to sexual and gender-based violence |  |
|  |  | Conflict analysis (tracking of media and other informant reports) |  |
|  |  | Other: ***(write in the column to the right)*** |  |
| 4 | **Food security and feeding practices** | Household livelihoods, resilience and coping, food access, food consumption, and feeding practices survey |  |
|  |  | Agricultural production monitoring |  |
|  |  | market analysis |  |
|  |  | household focus groups |  |
|  |  | risk assessment of desk-based food security |  |
|  |  | Other: ***(write in the column to the right)*** |  |
| 5 | **Nutritional status** | Repeated anthropometric sampling from sentinel communities |  |
|  |  | Anthropometric survey |  |
|  |  | Trend analysis from community-based or facility based anthropometric screening, and community-based management of acute malnutrition admissions |  |
|  |  | Desk-based nutritional risk assessment |  |
|  |  | Other: ***(write in the column to the right)*** |  |
| 6 | **Communicable diseases** | EWARN for epidemic alert and response |  |
|  |  | Other: ***(write in the column to the right)*** |  |
| 7 | **Non-Communicable diseases** | Survey to measure point prevalence of chronic diseases or retrospective prevalence of acute disease syndromes |  |
|  |  | Analysis of facility-based morbidity and mortality data |  |
|  |  | Other: ***(write in the column to the right)*** |  |
| 8 | **Morbidity data** | Analysis of facility-based morbidity and mortality data |  |
|  |  | Desk-based disease risk assessment and situation analysis |  |
|  |  | tracing and tracking of people in need of treatment continuation |  |
|  |  | Other: ***(write in the column to the right)*** |  |
| 9 | **Service availability and functionality** | HeRAMS (with updated geographical database of facilities) |  |
|  |  | Who, what, where, when (4Ws) |  |
|  |  | Other: write in the column to the right) |  |
| 10 | **Service coverage** | Coverage survey (eg, vaccination, health services, or nutritional programme) – ***(Add details in the column in the right)*** |  |
|  |  | Comparison of actual programme outputs vs target beneficiaries – ***(Add details in the column in the right)*** |  |
|  |  | Other: ***(write in the column to the right)*** |  |
| 11 | **Service quality or effectiveness** | Analysis of HMIS data (eg, on cure rates) |  |
|  |  | Other: ***(write in the column to the right)*** |  |
| 12 | **Population mortality** | Community-based demographic surveillance |  |
|  |  | passive body count surveillance |  |
|  |  | Retrospective mortality survey (verbal autopsies as add-on to explore causes of death) |  |
|  |  | Census (post-war) and demographic modelling |  |
|  |  | capture–recapture analysis |  |
|  |  | indirect (model-based) estimation |  |
|  |  | Other: write in the column to the right) |  |

**Annex 2: COREQ (Consolidated Criteria for Reporting Qualitative Research) Checklist**


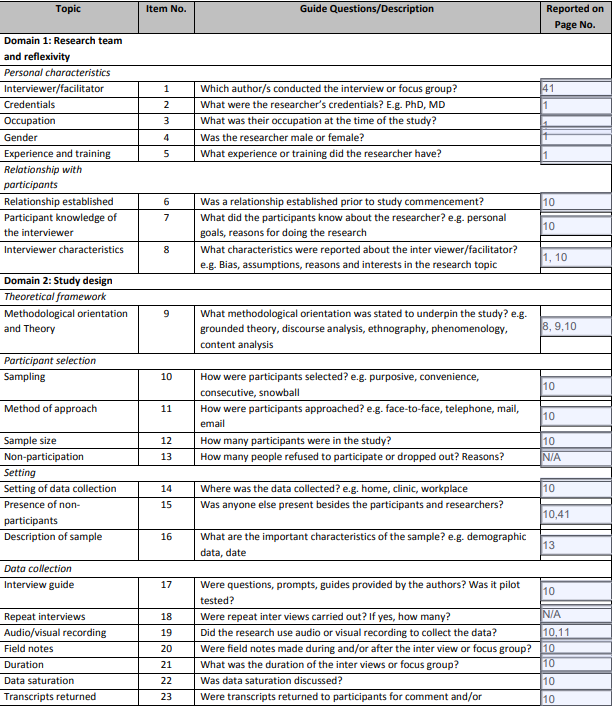

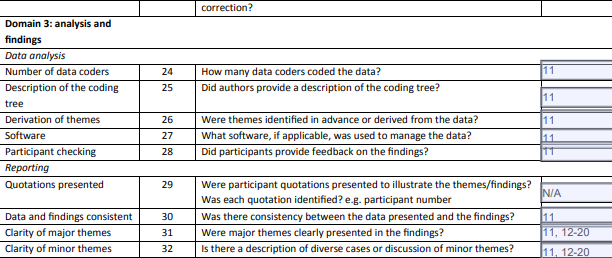


**Annex 3: Scoring Sheet for the Number and Quality of Available Public Health Information Domains in NWS**

| **Dimension**  **Score** | **Number of Available Sets** | **Data Completeness** | **Data Accuracy** | **Data Consistency** | **Data Frequency** |
| --- | --- | --- | --- | --- | --- |
| **0** | No dataset is available | Variables are not sufficient to calculate required or expected outcomes | Dataset does not match other available datasets or includes values that are unrealistic compared to what is known in the literature | Datasets are inconsistent and provide largely varying results across different facilities and/or sights in an unrealistic way | Data is entered once and never updated or rarely updated over the years. |
| **0.5** | 1-5 Datasets are available | Variable can be used to calculate some but not all required or expected outcomes | Dataset values the does not match other available datasets or includes values that are unrealistic compared to what is known in the literature, and outliers are not justified or explained | Datasets are inconsistent and provide largely varying results across different facilities and/or sights in an unrealistic way | Data is updated in a timely manner (monthly or yearly depending on the type of data) |
| **1.00** | 5 or more datasets are available | Variables can be used to calculate required or expected outcomes | Dataset variable match other available datasets or includes values that are unrealistic compared to what is known in the literature, and outliers are explained and justified | ___ | ___ |
